# Influence of graft vascularization on graft survival following homologous limbo-keratoplasty

**DOI:** 10.1007/s10792-022-02291-9

**Published:** 2022-04-05

**Authors:** Stefan J. Lang, Nicole Werner, Daniel Böhringer, Philip Maier, Thomas Reinhard

**Affiliations:** Eye Center, Medical Center, Faculty of Medicine, University of Freiburg, Killianstr. 5, 79106 Freiburg, Germany

**Keywords:** Limbo-keratoplasty, Keratoplasty, Limbal stem cells, Graft vascularization, Graft survival

## Abstract

**Purpose:**

Limbo-keratoplasty enables visual improvement and limbal stem cell transplantation at the same. During follow-up, most grafts show vascularization of the limbus. However, it is unclear whether vascularization is harmful due to immunologic effects or helpful to nourish the limbal stem cells and is therefore necessary for a clear graft. The aim of our study is to analyze the influence of graft vascularization on graft survival following homologous limbo-keratoplasty.

**Methods:**

In this retrospective study, we assessed all consecutive limbo-keratoplasties performed in our hospital. All eyes with suitable photo-documentation were included and divided into two groups (limbal stem cell deficiency and corneal dystrophy). We categorized the grade of vascularization (0, 1, 2, 3, 3b) and analyzed clear graft survival, recurrence of the underlying disease and the endothelial cell density (ECD) with regard to the reason for the graft. Event rates were estimated with the Kaplan–Meier method.

**Results:**

A total of 79 eyes with limbal stem cell deficiency and 15 with corneal dystrophies were analyzed. A high degree of graft vascularization had a tendency for better graft survival in limbal stem cell deficiency, whereas in corneal dystrophies, grafts with no vascularization had preferable outcomes. Recurrence-free graft survival was only seen in grade 1 and 3 vascularization in corneal dystrophies.

**Conclusion:**

Vascularization of the limbus seems to have an impact on the long-term outcome of limbo-keratoplasty. The effect seems to be favorable in limbal stem cell deficiency and on recurrence rates in corneal dystrophies. However, the latter might be overshadowed by an unfavorable immunologic effect in corneal dystrophies where the baseline immunologic risk profile is commonly more favorable than in limbal stem cell deficiency.

## Introduction

Allogenic limbo-keratoplasty was first introduced by Sundmacher et al. [1]. The aim of this surgical procedure is to restore the vision of the patient by grafting clear corneal tissue and to restore limbal stem cell function by grafting limbal tissueat the same time and within one graft [1]. Hence, this method of surgery poses a therapeutic strategy for diseases with corneal opacification and limbal stem cell problems. This includes bilateral limbal stem cell deficiency as a result of thermal or chemical burns or due to hereditary diseases like aniridia [2, 3]. Certain corneal dystrophies like granular or gelatinous corneal dystrophy are a result of a disease of the limbal epithelial stem cells [4, 5]. Therefore, limbo-keratoplasty is also used to treat certain corneal dystrophies [6–8] in order to avoid recurrences of the underlying dystrophy as it is known that following conventional penetrating or anterior lamellar keratoplasty recurrences of the respective dystrophy occur in almost 100% of cases.

It has been shown that fewer transplants develop a recurrence of the dystrophy if limbal tissue is also transplanted [9]. The simultaneous grafting of corneal limbal tissue is achieved by eccentric trephination of the donor tissue [1]. The resulting corneal button is then grafted centrally into the recipient tissue, usually with the limbal tissue on the upper rim to achieve protection by a location that is usually covered by the upper eye lid [1]. Despite being a promising therapeutic principle, allogenic limbo-keratoplasty has to be considered as a high-risk form of corneal transplantation. In contrast to conventional penetrating keratoplasty, not only corneal tissue is transplanted, but also limbal tissue which adds immunogenic potential to the transplant [10]. In order to improve the survival of the transplant, HLA (human leukocyte antigen) matching has been proposed [11]. Other treatment options to prolong the survival of the transplant and the limbal tissue include the use of topical steroids, systemic immunosuppression and the simultaneous grafting of amniotic membrane to protect the graft [3, 10]. Compared to other forms of surgical treatment for limbal stem cell deficiency, like keratoprosthesis, the survival of limbo-keratoplasty grafts is at least equal and there is the possibility of re-transplantation in the case of graft failure [10]. Ex vivo expansion of limbal stem cells or autologous limbal stem cell transplantation is not an alternative to limbo-keratoplasty as this technique is only used in cases of complete bilateral limbal stem cell deficiency where there is no possibility to harvest healthy autologous limbal stem cells. However, median graft survival of 3 to 4 years [10] still suggests room for improvement, possibly by means of improving the postoperative management. It is striking that a majority of limbo-keratoplasty grafts show vascularization of the grafted limbal tissue after transplantation. This limbal vascularization is superficial and different from the superficial or deep vascularization seen in case of graft failure. This exposes the graft to the host’s immune system and can lead to immune reactions, limiting the survival of the transplant [10]. On the other hand, the limbal tissue may need the vascularization in order to maintain the function of the limbal stem cells. Until now, there is no conclusion what has greater influence on the clear graft survival, the immunogenic aspects or the nourishing aspects of vascularization of the limbal tissue. Therefore, the aim of our study is to analyze the influence of graft vascularization on graft survival after homologous limbo-keratoplasty.

## Methods

**Fig. 1:**
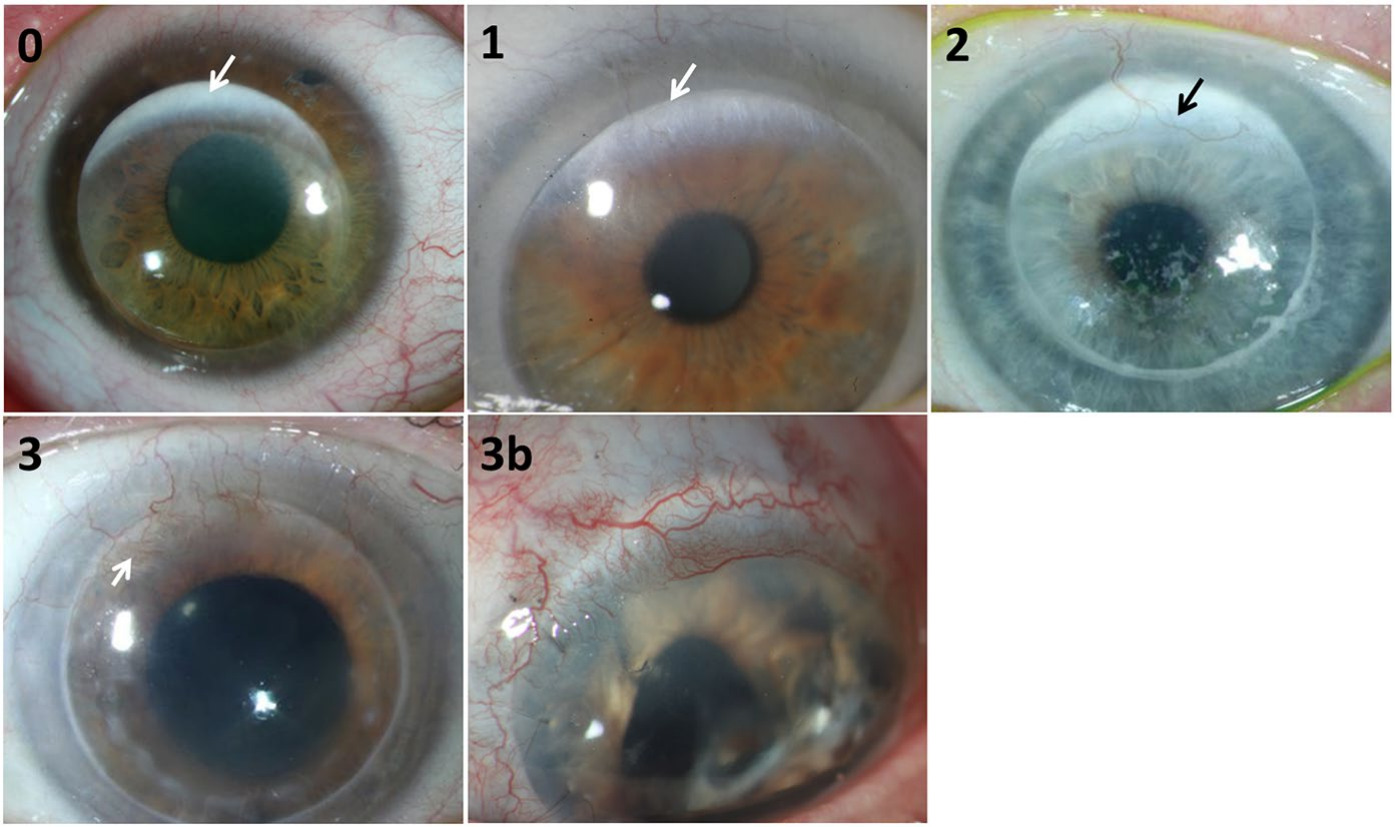
Grades of graft limbus vascularization: Grade 0: Grafts without vascularization. The limbal tissue is at the 12 o’clock position (arrow). Grade 1: One or two small vessels in the limbal tissue (arrow). Grade 2: 3 vessels with a vascular network (arrow). Grade 3: Complete vascularization of the graft limbus (arrow). Grade 3b: Additional hyperemia

The data of all consecutive limbo-keratoplasties performed at our institution from 2003 to 2020 were included in this retrospective study. All patients had topical and systemic immunosuppression if possible. Topical therapy usually consisted of dexamethasone eye drops. Systemic immunosuppression was usually a combination of cyclosporine and mycophenolate mofetil. In case of repeat transplantations in one patient, only the first one was included for analysis. We identified all cases with suitable photo-documentation of the graft that enabled to analyze the vascularization of the grafted limbal tissue. Only vascularization of the limbus was graded, not vascularization as indication to graft failure. The grade of vascularization was performed by two independent investigators according to example pictures of different grades of vascularization (Fig. 1). Grafts without vascularization were grade 0. One or two small vessels in the graft limbus were grade 1. Three vessels with a vascular network were grade 2. Grade 3 included grafts with a complete vascularization of the limbus and grade 3b had additional hyperemia. Both investigators were masked for the follow-up data at time of grading. Each transplant was categorized in one of two groups, depending on the underlying disease: limbal stem cell deficiency and corneal dystrophy. We assessed the overall clear graft survival, as well as recurrences of the primary disease due to limbal stem cell failure. The clear graft survival and the recurrence-free survival of the graft were estimated using Kaplan–Meier analysis for each group in consideration of the graft’s vascularization. We also assessed the corneal endothelial cell density and did a survival analysis with regard to grafts containing more than 1000 endothelial cells per mm^2^. Statistical analysis was done with R (R-project.org). Due to the retrospective exploratory character of our study along with the small sample size we decided to perform a descriptive presentation of the data rather than testing for statistical significance. Ethics committee approval was obtained at the ethics committee of the Albert-Ludwigs University Freiburg (Nr. 28/19).

## Results

A total of 206 eyes were assessed for this study. In total, 112 of those eyes had suitable postoperative photo-documentation. Ninety-four eyes did not have suitable photo-documentation, 79 with limbal stem cell deficiency and 15 with corneal dystrophies. All patients were treated with topical steroids. In the group of limbal stem cell deficiency, 89% were treated with systemic mycophenolate mofetil and 81% were treated with systemic ciclosporin. This results in a combination of both in 64% of the patients. In the group of corneal dystrophies, 82% were treated with systemic mycophenolate mofetil and 54% were treated with systemic ciclosporin. This results in a combination of both in 36% of the patients. Median age in the group with limbal stem cell deficiency was 51.9 years. The median age in the group with corneal dystrophies was 52 years. 43% of the patients were female. Median follow-up was 2.6 years in the group with limbal stem cell deficiency and 5.2 years in the group with corneal dystrophies. There were more eyes with a low grade of vascularization in the group with corneal dystrophies. There was no graft without vascularization in the group with limbal stem cell deficiencies (Table 1). There was a tendency to an increased vascularization in those cases that had multiple photo-documentations during follow-up (Table 2).

**Table 1:** Distribution of vascularization grades in both groups

Grade	Dystrophy (*n* = 29)	Limbal stem cell deficiency (*n* = 83)
0	28% (8)	0% (0)
1	21% (6)	6% (5)
2	28% (8)	12% (10)
3	17% (5)	36% (30)
3b	7% (2)	22% (18)
Not assessable	0% (0)	24% (20)

**Table 2:** Change of vascularization during follow-up

Change	Dystrophy (*n* = 10)	Limbal stem cell deficiency (*n* = 21)
Decrease (-1)	20% (2)	14% (3)
Without change (± 1)	30% (3)	24% (5)
Increase (+ 1)	50% (5)	43% (9)
Not assessable	0% (0)	19% (4)
Follow-up (years)	5,25/7,00/9,50	3,00/3,00/6,00

Preoperative visual acuity was 0.1 (decimal, quartiles: 0.05; 0.2) in the corneal dystrophy group and 0.009 (decimal, quartiles: 0.005; 0.05) in the limbal stem cell deficiency group. Postoperative visual acuity was 0.32 (decimal, quartiles: 0.07; 0.57) in the corneal dystrophy group and 0.01 (decimal, quartiles: 0.005; 0.1) in the limbal stem cell deficiency group.

**Fig. 2:**
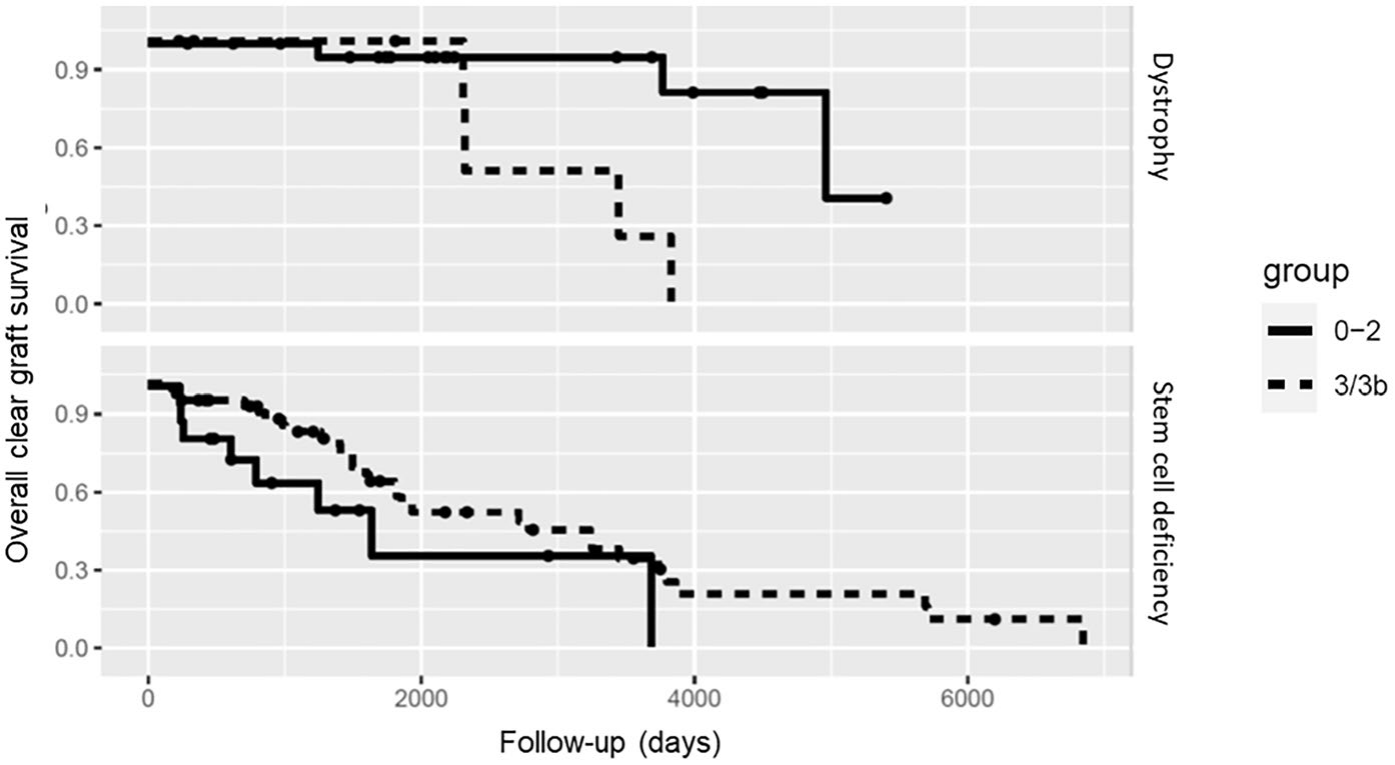
Kaplan–Meier plot for clear graft survival for limbal stem cell deficiency and corneal dystrophies. The groups were summarized in low vascularization (group 0–2) and high vascularization (group 3 and 3b)

After 2.5 years, 80% of grafts in the group with limbal stem cell deficiency were still clear. However, after 19,1 years no clear graft was documented. Grafts with vascularization grade 3 showed the longest clear graft survival, grade 2 had the shortest clear graft survival, and grade 1 and 3b were inbetween (Fig. 1). Statistical analysis showed no statistical significance (*p* = 0.1). Corneal dystrophies showed longer clear graft survival in general with 90% clear graft survival after 5 years. All grafts with grade 0 remained clear during complete follow-up. The graft survival decreased in the groups with higher vascularization with the lowest in grade 3 and 3b with no remaining clear grafts after 11 years (Fig. 2).

**Fig. 3:**
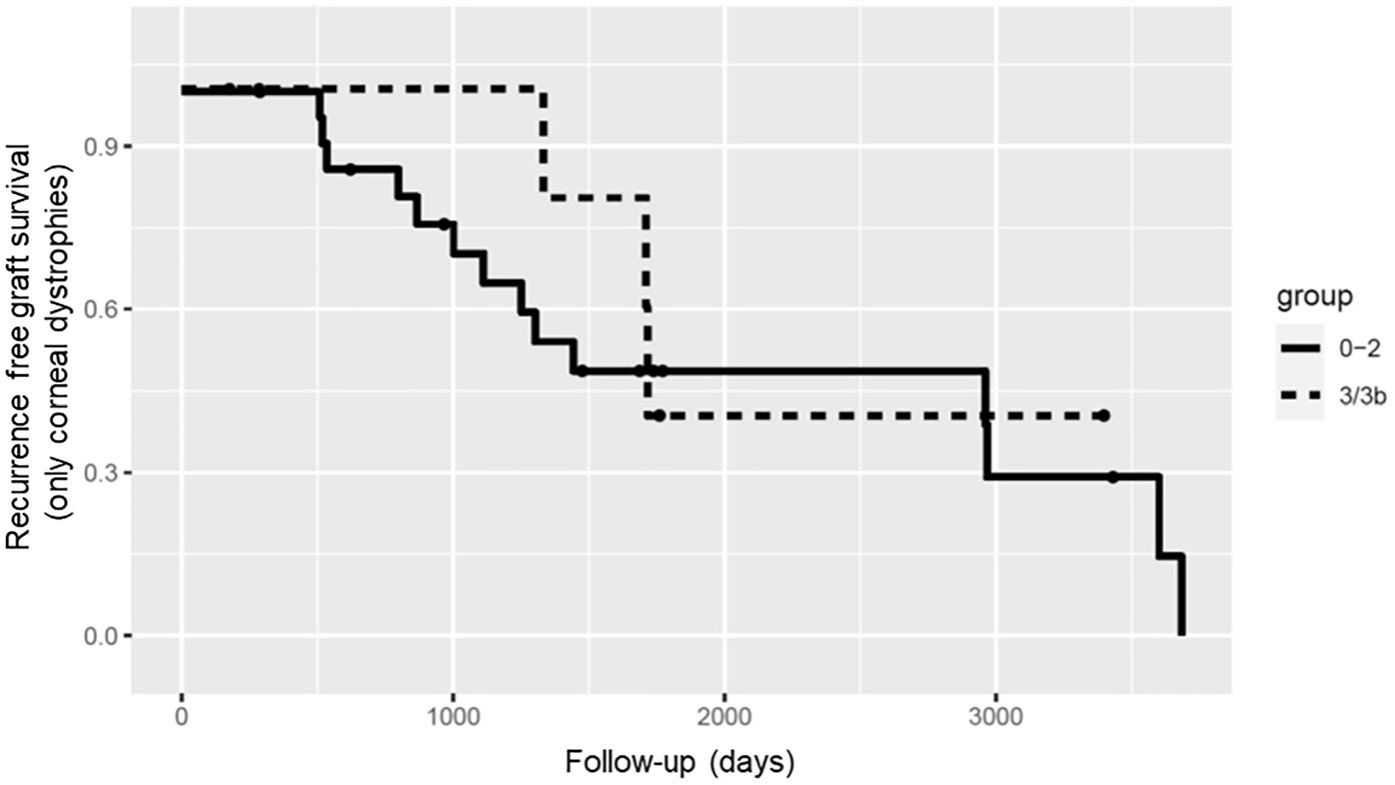
Kaplan–Meier plot for recurrence-free graft survival in corneal dystrophies. The groups were summarized in low vascularization (group 0–2) and high vascularization (group 3 and 3b)

Regarding recurrence-free graft survival in corneal dystrophies, only grade 1 and 3 included grafts that showed no recurrence during follow-up, whereas all the other grades showed recurrence in all grafts after 5 to 10 years (Fig. 3).

**Fig. 4:**
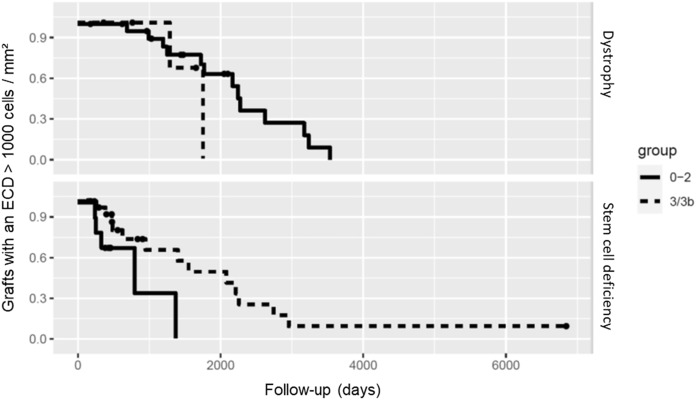
Kaplan–Meier plot for grafts with endothelial cell density higher than 1000 cells/mm^2^. The groups were summarized in low vascularization (group 0–2) and high vascularization (group 3 and 3b)

Regarding endothelial cell count, in the group with limbal stem cell deficiency, grade 2 and 3 vascularization showed grafts (50% and 25%) that maintained more than 1000 endothelial cells per mm^2^. In corneal dystrophies, grade 3 vascularization also showed grafts that maintained more than 1000 endothelial cells per mm^2^. All the other grades of vascularization did not maintain a high endothelial cell count during follow-up (Fig. 4).

## Discussion

Compared to previous studies, our results show a comparable or slightly better overall graft survival [10, 11] for grafts following limbo-keratoplasty in patients with limbal stem cell deficiency or epithelial/stromal dystrophies. The group with corneal dystrophies showed an overall higher rate of graft survival than the group with limbal stem cell deficiency.

Regarding the graft survival in relation to the grade of vascularization of the grafted limbus, a difference between both indications was visible. In limbal stem cell failure, grafts with a higher degree of limbal vascularization tended to have a longer graft survival. In the group of corneal dystrophies, there was a tendency to a higher graft survival with no or low vascularization of the transplanted limbal tissue. The latter is in concordance with the literature where a higher degree of vascularization of the graft is usually associated with higher risk of graft failure due to immune reactions [12, 13]. It seems possible that the vascularization of the transplanted limbal tissue might prove useful in limbal stem cell failure, possibly by nourishing the graft and keeping the transplanted limbal stem cells functional, whereas in corneal dystrophies the limbal function might be less important until late follow-up. Here, the immunologic drawbacks from vascularization may outweigh the benefits from protecting the limbal portion of the graft despite systemic and topical immunosuppression in both groups.

The high recurrence rate of corneal dystrophy in our population seems rather early in comparison to the literature, where usually recurrence is seen later [14–16]. Grafts with a vascularization of the limbus (grade 1 and 3) did not show any recurrence after 10 years. Therefore, a sufficient grade of vascularization might be needed to maintain function of the grafted limbal stem cells. Since vascularization was not favorable in corneal dystrophies in overall clear graft survival, the immunologic effect seems to surpass the advantage regarding the recurrence of the respective dystrophy. Other treatment options for epithelial corneal dystrophies, like phototherapeutic keratectomy, should be considered as alternate treatment option, especially in early stages of the disease (ZITAT).

Regarding the corneal endothelium, there is a steady loss of endothelial cells in all grafts following limbo-keratoplasty independent of the vascularization grades in the corneal dystrophy group and in all but one in the group with limbal stem cell deficiency. Reports in the literature show that such a chronic loss of endothelial cells has to be expected and is well within usual range, with an median endothelial cell count of 1035 cells/mm^2^ in the study of Khattak and Nakhli [17] and 550 to 628 cells/mm^2^ after 10 years in the study of Lass et al. [18]. In our study, especially in the corneal dystrophy group, there does not seem to be a correlation with vascularization.

Limitations of our study lie in its retrospective design, the limited number of patients and the possible bias in selection, since we could only include cases that had adequate photo-documentation. The selection bias might have excluded cases with very early graft failure. Also, the follow-up is relatively short. This is due to the retrospective nature of the study. Patients that live far away from our clinic might have their regular examinations close to home. We evaluated the overall clear graft survival, because a clear distinction between an endothelial graft failure and a failure of the transplanted limbus is not always possible in retrospect. Also, a certain distinction between status post-graft rejection and chronic endothelial cell loss was not possible due to the retrospective design.

In conclusion, the vascularization of the transplanted limbal tissue seems to have an impact on the long-term outcome of the limbo-keratoplasty grafts. The effect seems to be favorable in limbal stem cell deficiency and on the recurrence in corneal dystrophies. However, the latter might be overshadowed by an unfavorable immunologic effect in corneal dystrophies where the baseline immunologic risk profile is commonly more favorable than in limbal stem cell deficiency. Future prospective studies are needed to verify these results and explore possible therapeutic applications.
